# Social Drone Sharing to Increase UAV Patrolling Autonomy in Pre- and Post-Emergency Scenarios

**DOI:** 10.3389/frobt.2022.820239

**Published:** 2022-04-04

**Authors:** Isabella-Sole Bisio, Luca Morando, Carmine Tommaso Recchiuto, Antonio Sgorbissa

**Affiliations:** DIBRIS Department, University of Genova, Genova, Italy

**Keywords:** unmanned aerial vehicle (UAV), teams of drones, cloud architecture, path-planning, autonomy

## Abstract

Multirotor drones are becoming increasingly popular in a number of application fields, with a unique appeal to the scientific community and the general public. Applications include security, monitoring and surveillance, environmental mapping, and emergency scenario management: in all these areas, two of the main issues to address are the availability of appropriate software architectures to coordinate teams of drones and solutions to cope with the short-term battery life. This article proposes the novel concepts of Social Drone Sharing (SDS) and Social Charging Station (SCS), which provide the basis to address these problems. Specifically, the article focuses on teams of drones in pre- and post-event monitoring and assessment. Using multirotor drones in these situations can be difficult due to the limited flight autonomy when multiple targets need to be inspected. The idea behind the SDS concept is that citizens can volunteer to recharge a drone or replace its batteries if it lands on their property. The computation of paths to inspect multiple targets will then take into account the availability of SCSs to find solutions compatible with the required inspection and flight times. The main contribution of this article is the development of a cloud-based software architecture for SDS mission management, which includes a multi-drone path-optimization algorithm taking the SDS and SCS concepts into account. Experiments in simulation and a lab environment are discussed, paving the path to a larger trial in a real scenario.

## 1 Introduction

Unmanned Aerial Vehicles (UAV), in particular multi-rotor drones, have become increasingly popular in recent years. This is due to their versatility and, consequently, the fact that they can be employed in many different fields: from cinematography (Mademlis et al., 2019) to search and rescue (Tomic et al., 2012; Recchiuto and Sgorbissa, 2018a), from sport and leisure (Wang et al., 2017) to monitoring of power plants (Zormpas et al., 2018; Morando et al., 2022), just to mention a few. It is not difficult to imagine a near future when everyone will own one or more of these “devices”. Currently, researchers are exploring the possibility to use multiple drones to work in a team, where each drone is provided with artificial intelligence (AI) and communication capabilities: in this way, instead of being remotely operated, each drone might manage the tasks assigned in complete autonomy while interacting and exchanging information with team mates and with a ground station where missions are orchestrated.

Along this line of research, this work focuses on the development of teams of drones that will periodically monitor areas of interest to prevent or mitigate the impact of environmental hazards. Monitoring may be key in the pre-event phase, when the team periodically inspects a set of targets (e.g., for the early identification of hazards), or in the post-event phase (e.g., for damage assessment). The targets could be, for example, infrastructures to be kept under close observation before or after strong atmospheric events (bridge, roads, hospitals, schools, stacks of containers, etc.), fields or forests to prevent fires, soil slopes whose stability needs to be assessed before or after floods (Morando et al., 2020). As an aside, it shall be mentioned that there are still legal barriers to the development of teams of autonomous drones that operate “beyond visual line of sight” (BVLOS) since most countries allow only “visual line of sight” (VLOS) operations. However, international agencies such the European Union Aviation Safety Agency (EASA)1 and the Federal Aviation Administration (FAA) in USA2 are working towards a legal framework to allow BVLOS operations (for instance, FAA allows you to apply for a waiver for BVLOS under special conditions). Without entering into the details of the current regulations, it is evident that there is an urgent need from the society to define a timeline for autonomous flight regulations to come in force: such elements as the dimensions and weight of the UAV, whether they operate in populated areas or not, and the specific reason why autonomous flight is needed may play a key role in this process. Teams of small autonomous drones for periodic monitoring might become a reality soon, especially if they contribute to the safety of citizens and their operations are restricted to low population density areas.

In this scenario, one of the most significant limitations is the maximum flight time of drones and the consequent operational range (Tiwari et al., 2019), which is still very limited due to energetic constraints. Generally, a drone can operate for about half an hour, during which it may be complex to achieve all the mission objectives. As a feasible solution to this problem, this article builds upon the concept of Social Drone Sharing (SDS) proposed for the first time in (Morando et al., 2020), according to which citizens are welcome to contribute to the monitoring process. The key idea is that resident can volunteer to make their property available as Social Charging Station (SCS) to recharge drone batteries—in a spirit somehow similar to post-stations for changing horses of the pony-express delivery service.

To achieve this goal, the main contribution of this work is twofold:

• We developed and tested a new software architecture, which includes a cloud-server with associated services, designed to connect the three different actors contributing to a mission: 1) the Civil protection, 2) the resident volunteers, and 3) the drones;
• We implemented and tested an optimization algorithm based on the work proposed in (Sundar et al., 2016), aimed to find optimal paths for each drone, keeping into account the constraints of the SDS scenario.

Section 2 describes state-of the-art. Section 3 introduces the objectives and the methodology to achieve them. Section 4 describes the system architecture. Section 5 describes the algorithm for path computation. Section 6 reports about experiments performed in our laboratory. Finally, conclusions and future works are discussed in Section 7.

## 2 State of the Art

This section will briefly survey state-of-the-art concerning the use of UAVs for security, monitoring, and surveillance, the concept of “social drones” working in close interaction with people, and finally the cloud robotics paradigm.

### 2.1 UAVs for Security, Monitoring, and Surveillance

In the security, monitoring, and surveillance domain, there are several operations that multirotor drones can contribute to perform. A recently proposed application is detecting gas leakage (Tosato et al., 2019), where drones are employed to inspect and measure the gas level in areas inaccessible to humans. The same idea may be applied in all situations where the presence of pollutants, poisons, and chemical hazards or radiations might suggest using drones to avoid endangering the health of human workers (Chen et al., 2020).

However, the use of drones is not limited to situations in which human operators (e.g., firefighters, Civil protection, etc.) risk their lives. For example, in (Aznar et al., 2014) a catastrophic event is discussed where an oil tanker is the cause of a massive oil spill. Authors claim that a team of drones would be an excellent aid during such an emergency, helping humans quickly monitor the leak magnitude, and support search and rescue (SAR) operations to be conducted faster and more efficiently. Other examples included the use of drones to identify trapped people after hurricanes such as Katrina (Pratt et al., 2006) and Wilma (Murphy et al., 2008), or catastrophic seismic events such as L’Aquila (Italy) (Baiocchi et al., 2013) or Lushan (China) earthquake (Qi et al., 2016). Generally speaking, it is well-known that the time taken to reach the victims after a disaster can drastically change the impact of SAR operations (Qi et al., 2016): then, it is perfectly understandable that drones may play a key role to speed-up operations by acquiring relevant information whenever a quick intervention is required (Maza et al., 2010; Karaca et al., 2018; Lomonaco et al., 2018; McRae et al., 2019).

In disaster scenarios, the use of UAVs is not necessarily limited to SAR operations: pre- and post-event monitoring and assessment are crucial as well, and they may raise different challenges. Indeed, both pre-event and post-event operations may require repeatedly inspecting large areas since the situation may rapidly evolve. For example, consider periodically assessing the dryness of the vegetation in a peri-urban area to prevent fires or think about the actions to be taken for damage assessment after a severe hydrological event. In the first case, patrolling should be repeatedly performed to gather and update in real-time a representation of the pre-event situation; in the second case, periodic patrolling may be key to estimate the state of the involved areas and prevent further damages to people and things, or even assess and meet the primary and long-term needs of the communities affected by the disaster (Recchiuto and Sgorbissa, 2018b). Unfortunately, things become more problematic when drones are required to periodically monitor an area for a long time. In (Barmpounakis and Geroliminis, 2020), an experiment is described that has been conducted in the city center of Athens, in which a busy urban area is monitored by drones during peak hours, by repeating the same routine for a whole week. The authors of this work notice that one of the biggest challenges to address in periodic monitoring is the limited battery life of the drones, which cannot record videos for two and a half hours as the experiment would require it. Two options are then considered: the first is to swap drones with replacements while they are in the air to keep on monitoring the area. The second option is to replace drones batteries, which implies the presence of “blind intervals” of 10 min during which drones land, their batteries are replaced, they take off and come back to the point where they were before the pause. The lesson learned is that, whenever a mission requires drones to operate periodically for a long time, energetic constraints acquire the utmost importance.

Another emergency context in which drones can play a crucial role may be the delivery of first aid kits and life-saving medicines. Drones can achieve this in a shorter time than required by using land vehicles, for instance, in mountain areas or in the presence of heavy traffic. Think about an ambulance stuck in traffic: drones might be used to deliver a personalized kit, depending on the type of disease or injury, so that the victim can be assisted before the ambulance arrives (Sanjana and Prathilothamai, 2020). In (Bravo et al., 2016) it is proposed to use drones for first aid during outdoor sports, mainly when an unexpected health problem (e.g., heart attack, heatstroke, allergic reaction, dehydration) occurs in a remote site on the mountains or at sea. Finally, during the current COVID-19 emergency, the Italian police have used drones equipped with heat sensors to measure the body temperature of the people and send the information to authorities (The Local, 2020). But what if the time required to reach the intervention area turns out to be longer than the battery duration?

From all the examples above, it is evident that the limitations of multirotor drones concerning energetic autonomy may become very relevant in these contexts and deserve the greatest attention.

### 2.2 Social Drones

Personal drones are increasingly becoming part of our daily life: we expect that, in the near future, they will be employed for a considerable amount of routine tasks to support people (Cauchard et al., 2015; Floreano and Wood, 2015). In this general scenario, the “social drones” concept refers to drones flying close to users and interacting with them (Baytas et al., 2019).

To deal with the new challenges raised by social drones, in (Funk, 2018) the concept of “human-drone interaction” (HDI) is introduced. The authors claim that, as drones will have to interact more with human beings, their internal organization should not only be devoted to fulfilling their task: social drones need to have a human-centered design, which may imply different requirements compared with the usual hardware/software architectures for unmanned aerial vehicles. For example, parameters that affect human comfort and trust deserve the greatest attention in this process.

HDI studies have been conducted to understand better the behavioural standards that drones have to achieve while approaching humans. For example, the study described in (Cauchard et al., 2015) involves 19 participants who had to interact with drones: researchers observed that participants mostly applied the same kind of approach that is used with pets. In the work described in (Abtahi et al., 2017), commercial drones have been adapted to exhibit a safe-to-touch design by making use of propeller guards. This idea helps increase safety during flight and enables touch–based interaction, according to the hypothesis that, in public environments, people may have the necessity to physically interact with a drone controlled by someone else.

Other investigations have been done about the behaviours that drones should have in the presence of humans. In particular (Jensen et al., 2018), explored humans’ preferences about the distance at which people want to be perceived by drones, as well as the visual signals that drones should employ to communicate that they are aware of their human partners. Studies show that known methodologies for human-robot interaction (HRI) can not be just applied to HDI. The principal differences in these interactions are related to privacy and safety in public spaces (Brock et al., 2019).

Along this rationale, not only behavioural aspects have been studied, but also drone prototypes that may have a pleasant appearance for humans, with a design meant to enhance the sense of comfort while using them. Among the others (Yeh et al., 2017), explores social distance in HDI using a drone prototype with a “socially appealing shape”. The researchers added a cage around the drone to increase user comfort and a “friendly face” on the drone, partly inspired by the uncanny valley hypothesis that the sense of familiarity increases when the robot shares some similarities with a human (Mori et al., 2012). The study showed that this design choice reduced the acceptable human-drone distance during the interaction.

Among the possible applications of drones in everyday life, the research explored how drones might be useful as companions in a home environment (Karjalainen et al., 2017): the users appreciated using drones to simplify daily tasks such as housekeeping, cleaning, and fetching items. Similarly, some work proposed drones for taking selfies (Chen et al., 2015) and following people during jogging (Mueller and Muirhead, 2015), where the drone takes the role of a “jogging companion” flying alongside the runner. The potential of flying drones as navigation guides for pedestrians has been explored as well, as they might provide more direct guidance than the use of hand-held devices (Colley et al., 2017). Along this line, examples of social drones for visually impaired people have been discussed in (Avila et al., 2015; Avila Soto et al., 2017).

Finally, a study about people awareness about the use of drones in society is presented in (Tahir et al., 2019), by asking people such questions as “Are you familiar with drone technology and do you understand the term swarm of drones?” or “If a swarm of drones is used to monitor the area (e.g., cities, forests, farming fields, public events) and for gathering aerial images, would you be in favour of it or would you have any concerns?” The survey showed, among the other results, that 144 out of 187 participants had never or hardly ever used a drone, whereas only 43 subjects had used them for hobby or work. Concerning security, 115 participants had security concerns, 6 of them had privacy concerns, 8 had security as well as privacy concerns, and 1 participant added that any type of concerns was dependent on the functional capabilities of drones. Finally, 57 subjects were just a little worried or not worried at all.

To summarize, previous research shows that people are getting more used to drones, even if some concerns still exist and shall be taken into the highest consideration. People’s attitude towards drones is favourable enough to encourage their introduction in society and share with citizens the responsibility of using drones to improve the quality of life of the community in which they live.

### 2.3 Cloud Robotics

For the first time in 2010, James J. Kuffner adopted the term “cloud robotics”, referring to a robot equipped with an autonomous system that harnesses the cloud potential in all its aspects (Kuffner, 2010). Cloud robotics is made possible by advances in several fields, such as wireless networks, large-scale storage and communication technologies, and the Internet. According to this paradigm, robots may take advantage of the cloud for greater data processing and storage capability (Saha and Dasgupta, 2018).

One of the most relevant examples are Google autonomous cars, which connect to the internet to exchange a massive amount of maps and satellite images, which are merged with data retrieved by onboard sensors such as cameras, GPS, and 3D sensors. Each vehicle actively contributes to uploading information to the cloud about the situation just experienced so that the next vehicle that travels along the same road is updated with the most recent data. More generally, it has been shown that cloud robotics technology may have a wide application range (Saha and Dasgupta, 2018), including perception, navigation, manipulation, and natural language processing, in different domains such as manufacturing, service, social, agricultural, medical, and disaster robotics.

The work in (Waibel et al., 2011) provides a concrete example of an open-source platform that allow any robot equipped with a network connection to manipulate, generate, share, and reuse data. Specifically, the RoboEarth three-layered architecture emphasizes the concept that each robot should allow other robots to learn through its knowledge and vice-versa. The higher layer (cloud server) is the core of the whole architecture: it includes a database to store information that can be reused in several different scenarios (including images, point clouds, models, maps, object locations, but even semantic information associated to them through an ontology, action recipes, and skills) and provides access to this information through a portfolio of Web services. The middle-layer (generic components) provides software to implement typical robot skills and functionalities, such as action execution, environment modeling, semantic mapping, action and situation recognition, and labeling, learning. The lower level (robot-specific) interfaces the other two levels with the hardware-dependent functionalities of the robot via the so-called “skill abstraction layer”.

Other frameworks exist (Mohanarajah et al., 2015), e.g., with a specific focus on mapping (Riazuelo et al., 2014), dynamical allocation of resources (Hu et al., 2012), grasping (Kehoe et al., 2013) conversation (Recchiuto and Sgorbissa, 2020).

## 3 Social Drone Sharing: Objective and Methodology

A limitation to the widespread use of drones in emergency domains is the absence of true autonomy. The problem is twofold: not only autonomous navigation capabilities are missing in terms of guidance and localization, but also energetic autonomy is subject to severe constraints. Indeed, the ability to cover long distances without recharging batteries is usually limited to a flight time of 15–30 min, which may reveal insufficient to conclude the patrolling mission.

This article addresses pre-event and post-event intervention, two situations in which the Civil protection needs to periodically monitor different critical targets, hopefully in the shortest possible time. These targets could be, for instance: critical infrastructure (bridges, roads, hospitals, schools, stacks of containers, etc.) whose risk shall be evaluated or damage assessed before or after a severe hydrogeological event; fields or woods whose dryness shall be periodically assessed to prevent fires; soil slopes whose stability shall be monitored before or after floods. Unfortunately, even if drones could play a key role in the pre-event and post-event phases for monitoring and assessment purposes, the ability to periodically patrol and inspect targets over a vast area is still an open issue: in most cases, the maximum flight time may not be sufficient to visit all targets.

To overcome this limitation, we propose to involve citizens (also referred to as “resident volunteers” or just “residents” in the following) in the monitoring process by adopting the new concepts of Social Charging Stations (SCS), proposed for the first time in (Morando et al., 2020). The autonomy range of drones is extended through the SCS concept: residents may volunteer to take care of drones if they land on their property, thus contributing to overcome their energetic constraints.

**FIGURE 1:**
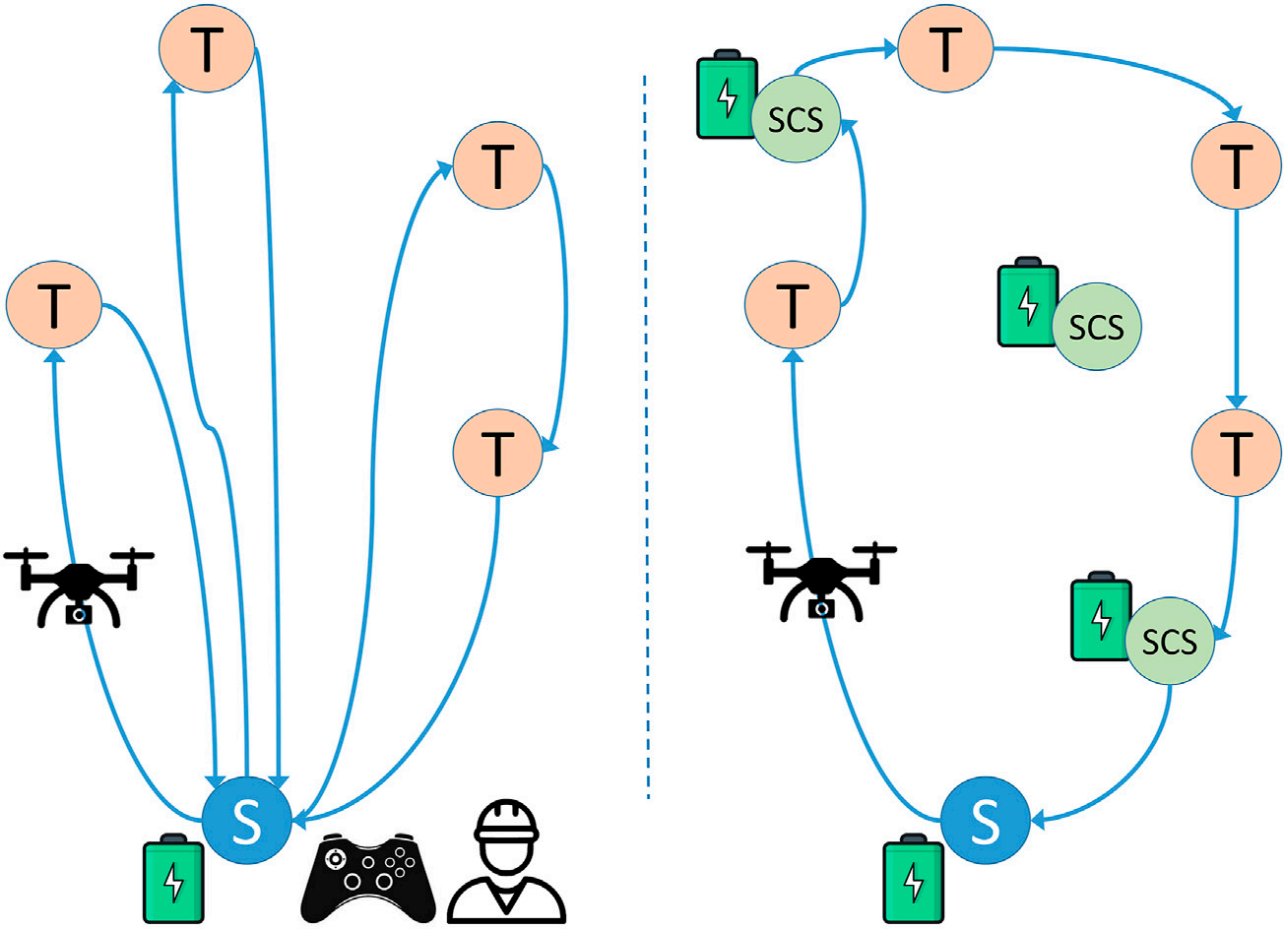
Left: A classical inspection methodology. Drones start from S and are remotely controlled to visit all targets (T), coming back to the starting point (S) when their batteries need recharging or replacing. Right: Social Drone Sharing (SDS) approach. Drones autonomously visit all targets, by landing at a Social Charging Station (SCS) whenever their batteries need recharging or replacing.

Figure 1 schematically represents the classical approach adopted for drone-based inspection (left) versus its “social” counterpart (right). The scenario on the left includes several targets (T) to visit and one take-off point (S), the only place where the drone can start, charge or replace its battery. Here, an operator remotely controls the drone [or a team of drones (Recchiuto et al., 2016)] through a radio command, and the drone’s path requires returning to the take-off point multiple times for recharging. In the scenario on the right there are additional Social Charging Stations (SCS) distributed in the area where targets are located. SCSs correspond to residents’ properties where the drone can be recharged or its batteries replaced: thanks to this, the drone returns to the starting point only after completing the inspection mission. Moreover, in the right scenario no operator is needed to control the drone: the path is automatically computed before take-off, after which the drone can fly autonomously to perform its mission.

**FIGURE 2:**
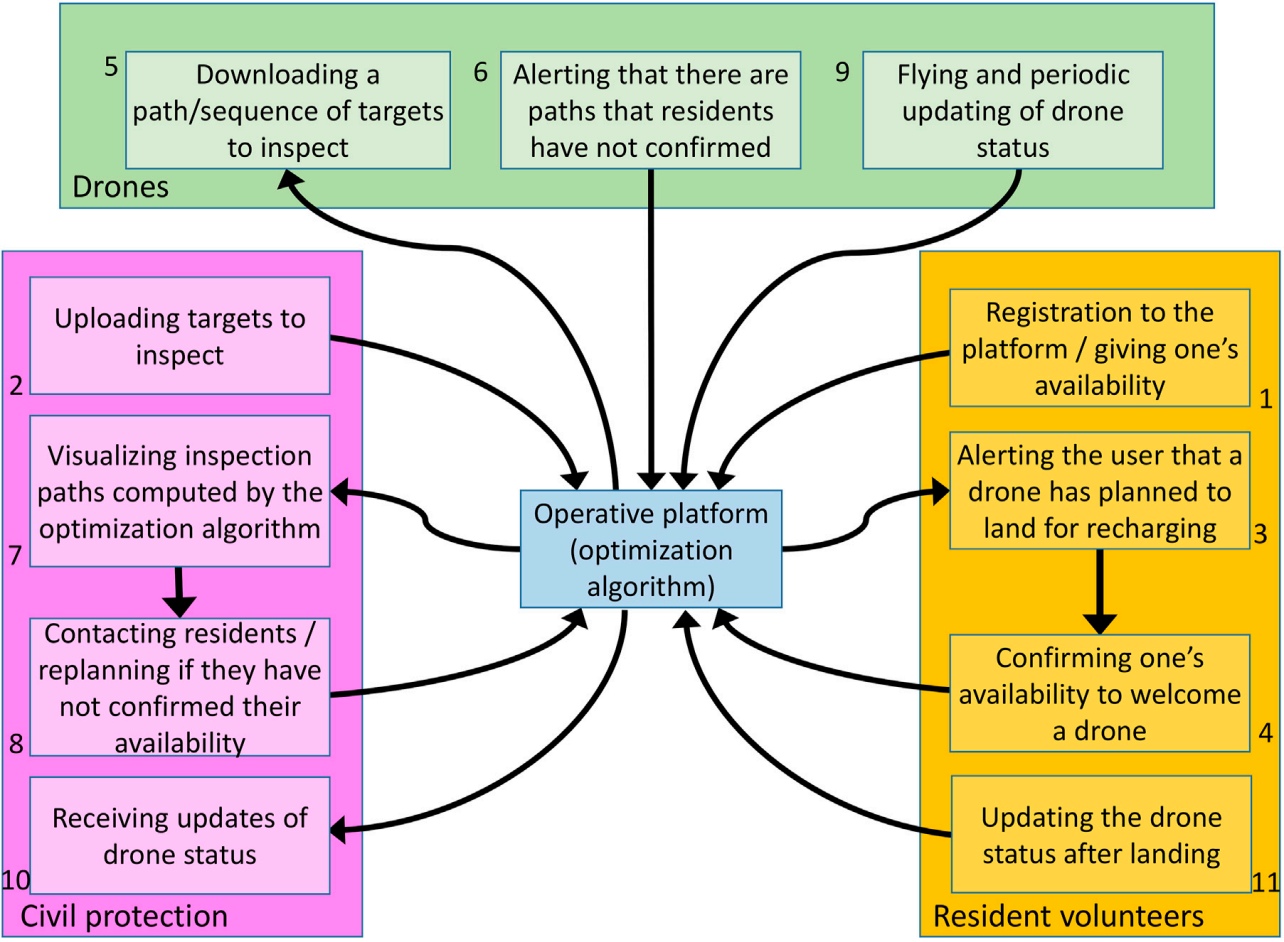
Actors in the cloud. 1: resident volunteers registers to the platform; 2: the Civil protection uploads targets; 3, 4: residents confirm their availability to welcome a drone; 5, 6: drones periodically check if there is a mission for them and alert the system if any residents along the path did not confirm their availability; 7, 8: the Civil protection checks if there are unconfirmed paths and possibly recompute them; 9: drones start the mission by periodically updating their status; 10: the Civil protection receives periodic updates about the mission status; 11: if a drone needs to be recharged, a citizen is alerted and updates the mission status after the drone has landed on their property.

Figure 2 focuses on the multiple “actors” interacting in our scenario: the Civil protection, resident volunteers and drones. Numbers in the Figure define the ideal sequence in which actors will perform the corresponding steps. First of all, resident volunteers shall connect to the cloud to give their availability to be part of the community as an SCS (1): they do this through an app, which will later alert them if their help is required to prepare the landing spot and recharge/replace the batteries of a drone. Thanks to this, residents are actively involved in the whole process, which may be particularly important especially in areas with low population density, where a community-based approach is repaid by greater safety. Civil protection authorities access the cloud to set the targets to survey depending on current needs (2). Then, they trigger a process for automatically computing optimal paths given all targets and the available SCSs in the area. Resident volunteers along planned paths are alerted that their SCSs will be needed soon: they are asked to confirm their availability to receive a drone for recharging (3, 4). As long as drones are switched on, they periodically connect to the cloud to check if a new mission is ready for them (5): if they have been assigned a path but some resident volunteers along the path have not confirmed their availability, drones alert the system that there are path segments that have not been confirmed; otherwise drones download the path and take-off. If some paths are not confirmed within a given deadline, the Civil protection may contact residents or recompute paths (7, 8). After a drone has started its mission, it periodically send updated information about the status of the mission and themselves, e.g., its position, battery status, etc. (9); the Civil protection receives periodical updates about all drones (10). Finally, when a resident volunteer is visited by a drone that needs recharging, it confirms that the drone has safely reached the SCS, takes care of it, and updates its status after the drone is ready to take-off again (11).

Notice that, in a real scenario, some of the steps above will performed in parallel or periodically repeated, also due to the fact that the Civil protection can add new targets to be inspected at any time, and new resident volunteers and drones can dynamically enter the system.

## 4 Cloud Implementation

In this section, we describe the architecture of the system and the services accessed by the three actors involved in the process.

### 4.1 System Architecture

As shown in Figure 2, all information exchanges are performed by three actors involved in the operation: the Civil protection, the resident volunteers, the drones. Each of these actors has specific tasks to complete and contributes to the mission in a unique way. To enable information exchange, the cloud server and its services are developed according to the REST API architectural style and the Flask micro-framework, using a virtual machine on the Microsoft Azure cloud. The server is associated with a database containing tables filled with various information. In the current implementation, an SQLite database is used.

Specifically, each service performs a query to a table of the database, summarized by the methods PUT, GET, PATCH, DELETE: PUT implements an action to write into a database table; GET implements an action to read from a database entry; PATCH implements an action to update the value in a database entry; DELETE implements an action to delete an entry in a database table. Please, observe that the PUT, PATCH, and DELETE methods only send data from a client to the server, whereas the GET method also returns the required information from the server to the client. Thus, the cloud server essentially works as an online information exchange platform between the different parties involved in the mission.

Finally, notice that this article ignores problems related to real-time drone navigation, which requires to implement proper algorithms for localization and control to move along the path taking into account the drone dynamics and environmental conditions, as well as strategies to inspect targets using on-board sensors that may vary depending on each mission. All this aspects are typically addressed by the on-board flight controller that commercial drones are provided with, supplemented by an additional on-board processor that performs high-level operations required to manage the mission and connect to the cloud to access its services. To know more about the strategies we plan to apply for autonomous navigation, possibly ensuring accurate localization, and obstacle avoidance when a target to be inspected has been reached, please refer to our previous work (Piaggio et al., 2001; Iacono and Sgorbissa, 2018; Morando et al., 2022).

### 4.2 Service Implementation

Here the implemented services are explained in detail, without providing details whether they are implemented as GET, PATCH, DELETE, or PUT methods. To this end, in the rest of the article, we assume a graph that comprises a set of SCS and target nodes connected through links: SCS nodes are labelled with the coordinates of a resident volunteer’s house where drones are allowed to land, whereas target nodes are labelled with coordinates of an area that a drone should visit and inspect. This concept will be better formalized when we introduce the algorithm for finding optimal paths in Section 6.3.

**FIGURE 3:**
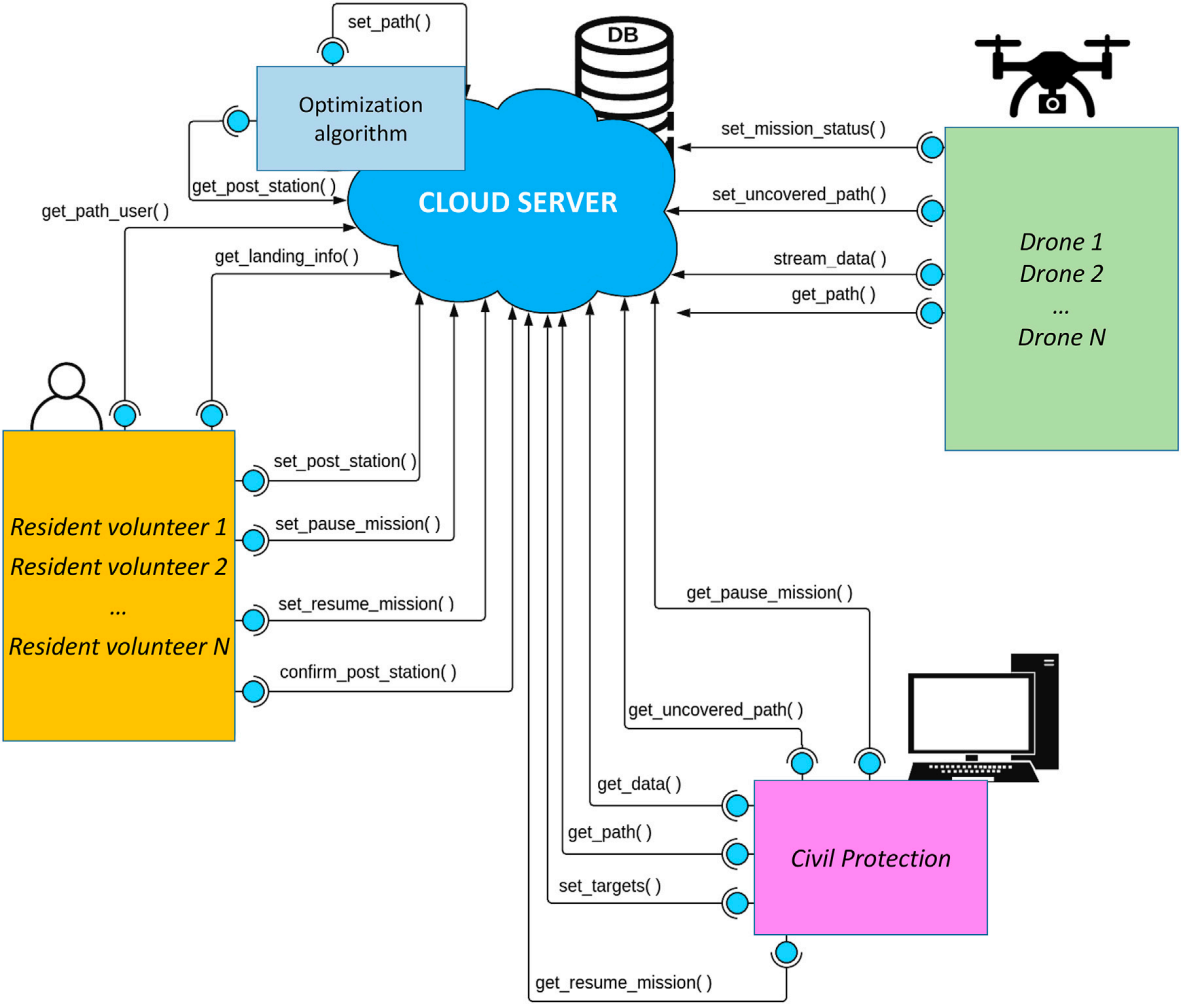
Cloud-based architecture with actors (Civili protection, residents volunteers, drones) and web services implemented.

All the services described below are listed to match the order in which they are called in Figure 2. They are also depicted in the software architecture structure in Figure 3, by reporting the three actors and the services that they typically use. All the returned values, if any, are encoded in a JSON file.

• set_post_station(first_name, last_name, address, city, state, zip_code, user_coords, availability_from, availability_to). This service is called by the resident’s app in step 1 when they give their availability to be part of the community. It requires inserting the resident’s personal data, address, GPS coordinates of the house, and the time range in which the resident is available to take care of a drone. If residents sign up to the system, the algorithm for path computation will consider their property as a potential SCS while building the graph of the target and SCS nodes. It returns a user ID that will be later used to check if there are drones to take care of.
• set_target(targets, target_coords, target_insp_times). This service is called by the Civil protection in step 2 to set targets. Input parameters are the number of targets to be visited, their coordinates, and the inspection time that the drone should spend on each target. After this, the service activates the functionalities to start computing paths.
• get_post_station(). This service is mostly called from within the path computation algorithm. No input parameters are needed since this function is in charge of reading the SCS coordinates directly from the database, previously uploaded by each resident when signing up to the system.
• set_path(drone_ID, path_coords, mission_from, mission_to, covered_path, uncovered_path, uncovered_from, uncovered_to, mission_status). This service is automatically called at the end of the path computation algorithm. The main input parameters are the drone ID, the coordinates of all nodes along the path, the time range of the mission, the covered and uncovered paths (covered/uncovered paths are segments of the whole path for which residents did/did not gave a confirmation). A drone can take off as soon as all the SCSs along its path have been confirmed by residents. On the other side, the residents who signed up to the system will receive a message: they need confirming that they will be available in the time frame when a drone is expected to land on their property.
• get_path_user(user_ID, user_coords, request_from, request_to, request_status). This service is periodically called by residents in step 3 to check if any drones are planning to land on their property. The main input parameters are the user ID and GPS coordinates. It replies with the ID of drones that have scheduled the resident’s house as an SCS along their path, if any.
• confirm_post_station(user_ID, drone_ID, index). This service is called by resident volunteers in step 4 to confirm or not their availability to take care of drones that need landing on their property. The main input parameters are the user ID, the drone ID, and the index of the path segment leading to the resident’s property. Once all the segments of the path have been confirmed, the mission status of a drone changes to *I can take off*, thus allowing it to start its mission.
• get_path(drone_ID). This service is periodically called by drones (and, if needed, the Civil protection) in step 5 to download a path assigned to the drone with the corresponding ID. If all residents along the path confirmed their availability to welcome the drone, the latter can take off and immediately jump to step 9.
• set_uncovered_path(drone_ID, index). This service is called by a drone in step 6 if it has been chosen for a mission but it can not take off since not all segments of the path have been confirmed by resident volunteers. The input parameters are the drone ID and the index of the missing path segment.
• get_uncovered_path(drone_ID). This service is called by the Civil protection in step 7 to check if any drone has a path still requiring confirmation from the residents. If residents do not give their confirmation within a given time, this may trigger an alert or possibly require to execute the algorithm for path computation again, step 8.
• set_mission_status(drone_ID). This service is called by drones in step 9 to update the mission status of a drone with a given ID, which could be one of the following: *I can not take off*: in case no paths have been assigned to that drone or the path has not been confirmed yet; *I can take off*: if there is a path that has been assigned to that drone and all the resident volunteers along that path confirmed to be available as SCSs; *I am in pause*: this state is explicitly set by a resident volunteer that is currently managing a drone landed on his/her property to recharge/replace its battery; *I can resume the mission*: this state is explicitly set by a resident volunteer when the battery has been recharged/replaced has ended and the drone can continue its mission.
• stream_data(drone_ID, status, landing_status, battery, drone_coords). The drone periodically calls this service to update the database with information about itself in step 9. The input parameters are the drone ID, any valuable information about its status (including acquired sensor data), the mission status, the battery level, and its position.
• get_data(drone_ID): this service is called by the Civil protection in step 10. It returns any valuable information about the drone including the mission status as well as the drone current position and remaining battery.
• get_landing_info(drone_ID). This service is periodically called in step 11 by a resident volunteer that confirmed his/her availability to welcome a drone with a given ID, in order to know its estimated arrival time.
• set_pause_mission(user_ID, drone_ID). This service is called in step 11 by a resident that, after a drone has landed, notifies that the drone has been taken care of and its batteries will be recharged/replaced soon. The input parameters of this function are the resident ID and the drone ID.
• set_resume_mission(user_ID, drone_ID). This service is called by a resident in step 11 when the drone has been recharged, and it can resume the mission. The input parameters of this function are the resident ID and the drone ID.
• get_pause_mission(drone_ID). This service is called by the Civil protection in step 10 to check if a drone with a given ID has been taken care of by a resident volunteer.
• get_resume_mission(drone_ID). This service is called by the Civil protection in step 10 to know if a drone with a given ID is ready to resume its mission.

**FIGURE 4:**
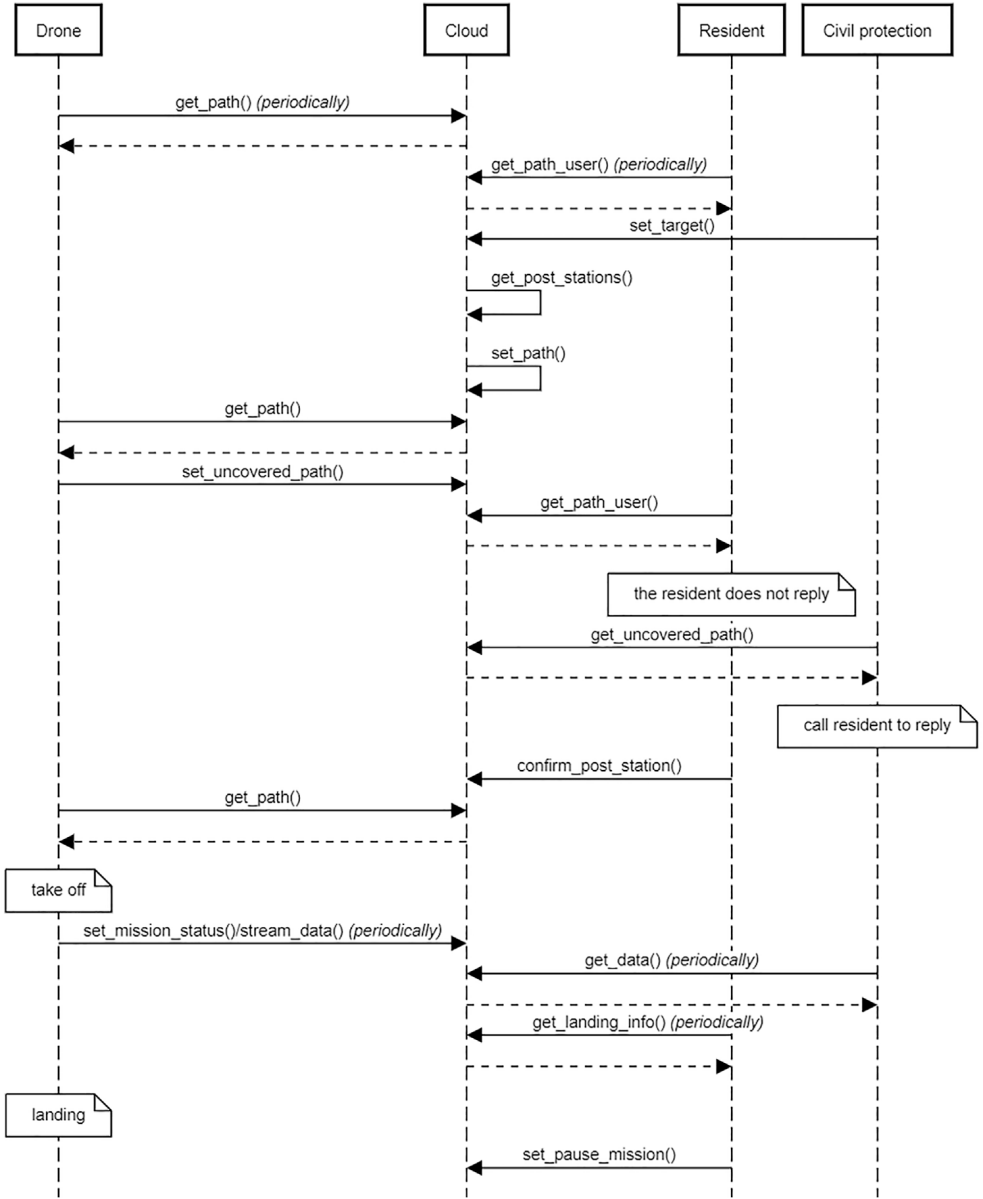
Sequence diagram representing the chain of events involving the Civil protection, resident volunteers and drones.

**FIGURE 5:**
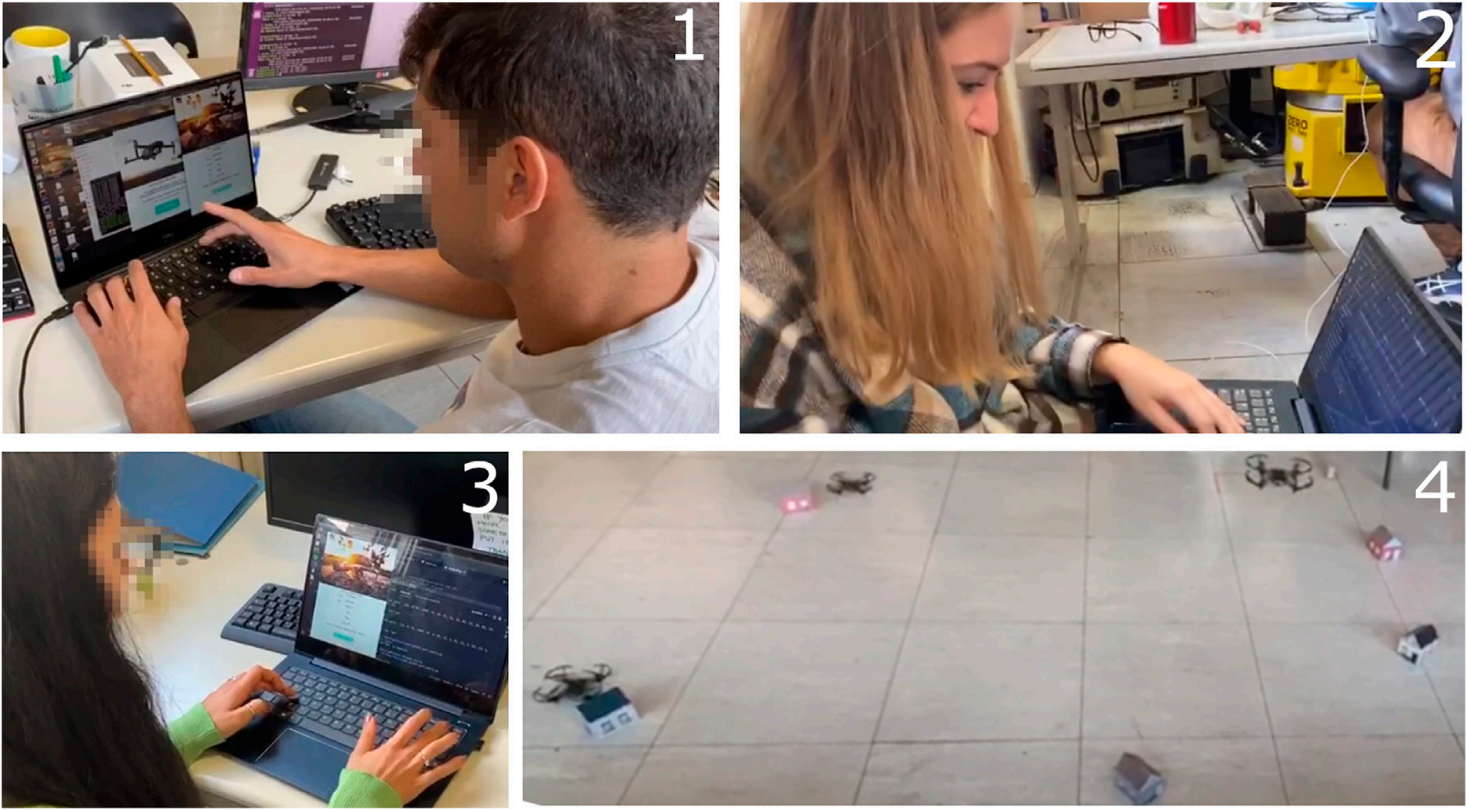
1: Resident volunteers subscribe to the system. 2: Civil protection insert a mission. 3: Resident volunteers confirm their availability for a given time range. 4: DJI Tello drones can take off.

The sequence diagram in Figure 4 shows the chain of events of a mission, also summarized in Figure 5. We assume that resident volunteers have already subscribed to the system. In the diagram, both the residents’ apps and drones periodically query the cloud to check if there are new paths (get_path, get_path_user). At some point, the Civil protection uploads a mission, described as a set of target locations to be visited (set_target): this triggers the procedure for path computation, whose output is a set of paths that drones should execute (set_path). Drones realize that there are new paths but some path segments have not been confirmed yet, and notify this information to the cloud (set_uncovered_path). Similarly, resident volunteers are notified that there are paths: however, they do not immediately confirm their willingness to take care of drones, a situation detected by the Civil protection (get_uncovered_path) finally leading to all residents confirming their availability (confirm_post_station). Drones can take off and start sending periodic updates about their status (set_mission_status stream_data), which are periodically received by the Civil protection (get_data). Similarly, residents periodically query the cloud for information about incoming drones, to be ready to recharge them or substitute their batteries as soon as they land on their property (set_pause_mission).

## 5 Path Computation

This section introduces the path computation algorithm used to find the drones’ optimal paths (i.e., less battery consuming) that visits all targets by meeting all constraints, and discusses about its limitations.

### 5.1 Finding Paths in the Social Drone Scenario

The algorithm to find optimal paths runs on the cloud, taking advantage of the cloud computing power. This aspect is particularly relevant by assuming that more instances of the algorithm might be executed in parallel by different actors accessing the service (e.g., local Civil protection authorities operating with the support of local resident communities), thus improving performance and preserving the confidentiality of sensitive data—such as the drones’ current positions or the resident volunteers’ identity and house coordinates.

In the current version of the system, the algorithm used for path computation has been taken from a previous work described in (Sundar et al., 2016), formulated and implemented exploiting the optimization tools provided by software such as Gurobi3. The optimization problem we consider is called Multiple Depot Fuel-Constrained Multiple Vehicle Routing Problem (FCMVRP) and is solved through mixed-integer linear programming. According to the FCMVRP formulation, a set of targets, a set of depots, and a set of vehicles are given. Each depot initially hosts a different vehicle, and—according to the terminology used in this article—the depots may also play the role of SCS whenever a vehicle passes through them. Specifically, when a vehicle reaches a depot, it refuels to its full capacity. The FCMVRP objective is to find a feasible route for each vehicle, according to the following constraints:

• Each vehicle’s path shall start and end at the same depot;
• Each target shall be visited at least once by some vehicle;
• No vehicle shall run out of fuel as it traverses its path;
• The total cost of the routes for the vehicles shall be minimized.

Possible applications requiring to solve FCMVRP are path-planning for drones or routing for electric vehicles based on the locations of charging stations: however, there are some differences with our original formulation of the SDS problem, which we will outline in the next section.

In (Sundar et al., 2016), four different mathematical formulations are proposed to approach the problem: arc-based formulation, arc-based formulation with strengthened inequalities, node-based formulation, node-based formulation with strengthened inequalities. For this work, only the first formulation is considered and implemented. In order to formally describe the constrained minimization problem, the following terminology is adopted:

• *T* denotes the set of targets *t*_1_, … , *t*_*n*_;
• *D* denotes the set of depots (that play also the role of SCS) *d*_1_, … , *d*_*k*_;
• Each depot *d*_*k*_ initially hosts a vehicle v_*k*_;
• *G* = (*V*, *E*) is a fully connected directed graph, where *V* = *T* ∪ *D* is the set of edges and *E* is the set of arcs connecting neighbouring vertices in *V*;
• *G* does not contain self-loops (i.e., there are no edges departing from and arriving to the same vertex);
• Each edge (*i*, *j*) ∈ *E* is associated with a non-negative cost *c*_*ij*_ required to travel from vertex *i* to vertex *j* and the fuel *f*_*ij*_ spent by traveling from *i* to *j*;
• The cost required to travel from vertex *i* to vertex *j* is directly proportional to the fuel/battery charge used in traversing the edge (*i*, *j*), that is *c*_*ij*_ = *K f*_*ij*_ (*c*_*ij*_ and *c*_*ji*_ may be different, but for the purpose of this paper, we assume *c*_*ij*_ = *c*_*ji*_);
• The travel costs satisfy the triangle inequality: for every *i*, *j*, *k* ∈ *V*, *c*_*ij*_ + *c*_*jk*_ ≥ *c*_*ik*_;
• F denotes the maximum fuel/battery capacity of the vehicles.

Each edge (*i*, *j*) ∈ *E* is associated with a variable *x*_*ij*_, which equals 1 if the edge (*i*, *j*) is chosen to be part of a path, and 0 otherwise. Also, each edge (*i*, *j*) is associated with a flow variable *z*_*ij*_ which denotes the total fuel/battery charge consumed by a vehicle as it starts from a depot in the vertex *j* when the predecessor of *j* is *i*.

Using this notation, the minimization problem can be described as follows:

$$minimize\underset{\left(i,j\right)\in E}{\sum }c_{ij}x_{ij}$$ (1)

subject to:

$$\underset{i\in V}{\sum }x_{di}=\underset{i\in V}{\sum }x_{id}\;\;\forall d\in D$$ (2)

$$\underset{i\in V}{\sum }x_{ij}=1\;and\;\underset{i\in V}{\sum }x_{ji}=1\;\;\forall j\in T$$ (3)

$$\underset{j\in V}{\sum }z_{ij}-\underset{j\in V}{\sum }z_{ji}=\underset{j\in V}{\sum }f_{ij}x_{ij}\;\;\forall i\in T$$ (4)

$$0\leq z_{ij}\leq Fx_{ij}\;\;\forall \left(i,j\right)\in E$$ (5)

$$z_{di}=f_{di}x_{di}\;\;\forall i\in T,\;d\in D$$ (6)

$$x_{ij}\in \left(0,1\right)\;\;\forall \left(i,j\right)\in E$$ (7)

The constraint 2 says that the number of arcs that are directed towards a depot should be equal to the number of arcs that leave from the depot towards other directions. It also implies the number of drones entering a depot has to be equal to the number of drones leaving the depot and that drones must return to their departure point to close the path. The constraint 3 expresses the fact that there must be only one arc that reaches the target and only one that leaves the target. In other words, each target must be visited only once. The 4 is a connectivity constraint and eliminates the target sub-tours. It considers all possible paths that a drone has made to get up to a certain point: the total fuel/battery consumption is equal to all the costs to travel the last sub-path portion plus the total cost that is spent to get to that point. Eqs. 5, 6 ensure that the fuel/battery charge consumed by the vehicle to travel up to a depot does not exceed its maximum fuel/battery capacity F. The last constraint 7 is the binary restriction on the variables: either an edge is present or not present in the path.

### 5.2 Limitations in the Social Drone Sharing Scenario

As mentioned above, the optimization algorithm does not perfectly match the requirements of our scenario. Two main differences exist that should be re-considered in future works.

The first difference is that the problem formulated in (Sundar et al., 2016) requires each drone to perform a closed path: each drone has to depart from and return to the same depot, which in our work correspond to a SCS. However, in our scenario, a drone can start from a resident volunteer’s house and end its path at another resident volunteer’s house, different from the one where it started. The difference is due to the fact that, in our scenario, drones are used to perform periodic inspections and not to deliver parcels, which would require them to come back to their depot to reload their stocks periodically. Suppose that a drone has landed at an SCS after completing a mission and the Civil protection needs to perform a new mission. Additionally, suppose that the new targets to inspect are close to the targets just inspected, i.e., before landing at the SCS for recharging. Then, it would be convenient to leave the drone at its current SCS, waiting for new instructions rather than coming back to the place from where the drone originally comes.

The second difference is that, in FCMVRP, the term depot and SCS are considered as synonyms, i.e., there are no SCSs that are not also depots. So then, for the computed paths to be executable, FCMVRP requires that all SCSs must be initially provided with a drone. This is not perfectly coherent with our formulation of the problem, where only a subset of the SCSs will likely host a drone recharging its batteries at a given time. That is, the concept of depot and SCS should be decoupled. By exploiting the cloud architecture, this difference is currently addressed by involving the different actors interacting with the system as follows. Once the path computation algorithm returns a set of candidate paths that visits all targets, each path needs to be individually re-considered by the Civil protection and confirmed before being assigned to drones. Suppose that the path passes through some SCSs: by manually inspecting the path, the Civil protection will have to select the SCS to be considered as a depot (i.e., a starting point hosting a drone) depending on the actual position of all drones, whereas the other SCSs along the path will only play the role of charging station.

**FIGURE 6:**
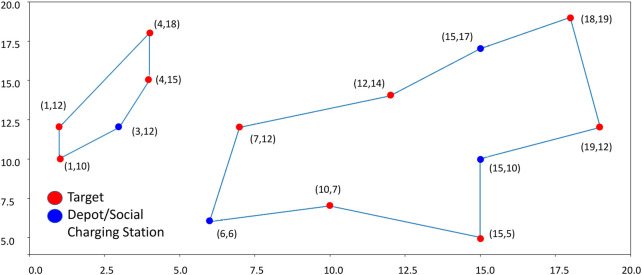
Example of algorithm output. Red circles correspond to targets to be visited by drones; blue circles correspond to Depots/Social Charging Stations.

This concept is shown in Figure 6. The left path does not leave many choices since there is only an SCS in position (3, 12) (blue circle) that can be selected as a depot: the path can only start and end at that point. Instead, the right path includes several SCSs that can be chosen as a starting point, respectively, in positions (6, 6), (15, 17), and (15, 10). If the SCS in (6, 6) is chosen as the depot (which means that it hosts a drone), then the SCSs in (15, 17), and (15, 10) are simply used as charging stations. Please notice that, when doing this, it may happen that a feasible solution to the problem cannot be found if none of the SCSs along a path can play the role of depot: think about what will happen if, in the previous example, there is not a drone in (3, 12). Solutions to deal with this situation can be implemented, for instance, by exploring different sub-optimal solutions to the problem producing a different set of closed paths.

## 6 Experiments

This section describes experiments in a laboratory setup to validate the software architecture and check that services are accessed by the three actors in the right sequence without making undesirable situations happen, measure the time required to access cloud services, and finally the time required for path computation under different conditions.

### 6.1 System Architecture and Services

The first phase is important to reveal any bugs or malfunctions in the developed services and to check whether the database is accessed as planned. To this aim, we simulate a sequence of events that would occur in a real scenario using a team of DJI Tello drones that are particularly convenient for experiments in a laboratory setup, Figure 5. Specific attention is paid to verify that each service properly fills the database with new data and updates/downloads existing data. This has been verified using different configurations of targets and SCSs. Precisely, the following steps are executed:

• Microsoft Azure cloud services are started. The cloud server is now active and listening to any potential requests through the REST web services.
• Drones are switched on and start periodically calling the proper service to check if they have been assigned a sequence of targets to inspect. They will take off only if this happens. Otherwise, they will remain in the current status and location. Notice that DJI Tello do not have an onboard PC, and therefore they are controlled by external PCs that connect to the cloud—this choice works well for laboratory experiments but shall be revised for a real-world scenario.
• Resident volunteers give their availability through the graphical user interface (GUI) of the app they are provided with. In a real case, this might require a complex subscription process to confirm the identity of residents, check how reliable they are, activate an insurance policy, and so on. On the opposite, during our tests, subscriptions are collected immediately before the drones start, using different computers connected to the cloud server that simulates different residents. Data entered by the residents are uploaded to the cloud database: from that time on, their coordinates can be used by the optimization algorithm as SCSs. The application that connects residents with the cloud server stays active in the background, ready to send them notifications if they are needed to welcome a drone whose path visits their house.
• The Civil protection uploads targets to be visited and runs the optimization algorithm. Targets’ coordinates correspond to areas to be inspected for a given time: the algorithm returns a set of candidate paths that solve the problem given targets and the available SCSs and drones. The Civil protection confirms the computed paths, the drone assigned to each path and its starting SCS.
• After paths have been confirmed, the residents whose coordinates have been chosen as SCSs confirm their availability in the time range when the drone is expected to land on their property. Specifically, the residents’ app connected to the cloud server, continuously checking for updates in the background, opens its GUI and displays a message inviting them to confirm that they agree to be visited by a drone for re-charging/replacing batteries.
• If all residents along a path respond positively to the mission, the drone takes off.

This whole pattern has been reproduced three times on ten different target/SCS configurations, for a total of 30 tests, without detecting any flaws in the process. Of course, this does not exclude the possibility of major problems emerging in the future, but paves the way to further development and to a larger trial with more residents and drones involved in a more realistic scenario.

### 6.2 Server Response Time

In the second phase, we measure the response time of the cloud server when subject to stress tests. Specifically, the objective is to calculate the time required to respond to the requests of different agents (drones and residents) by increasing both the number of connected agents and the frequency according to which services are called.

Please notice that, up to now, we have described the system in a simple scenario with a small number of resident volunteers and drones and a limited geographical area. However, the system might be used in significantly more complex scenarios: then, it is essential to estimate its behavior in advance when a higher number of requests are expected to occur (e.g., when several local Civil protection authorities, residents, or drones operating in different areas concurrently access the system). To make things worse, up to now we have hypothesized the case that the system is used in a pre- or post-event scenario, where a delay in executing a mission may not be very critical. However, the situation radically changes when employing the system in during emergencies. In this case, the perception of an imminent danger leads all actors involved to demand higher performances since a rapid response may be crucial, which the system needs to ensure.

During this test, requests to the cloud are made by 50, 150, 250, 350, 450 agents: each agent simulates a drone or a resident volunteer. To this end, each drone/resident is associated with a separate thread running on the same PC. Every thread makes a periodic request to the cloud, with an initial delay computed as a random number between 1 and 3 s to simulate asynchronous requests that would happen in a real scenario. Periodic requests coming from the same agent/thread occur with a period that varies in different experiments, i.e., 10, 1, 0.1, and 0.01 s.

**FIGURE 7:**
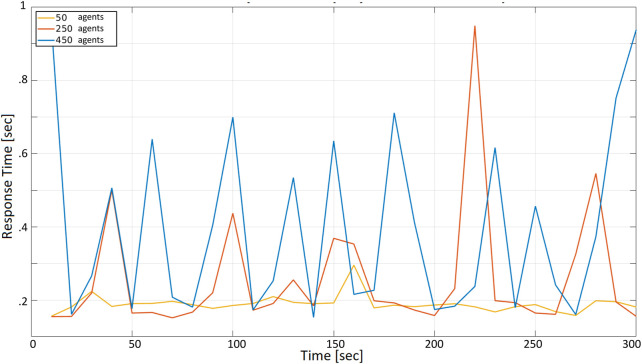
Cloud response time with a request period *r* = 10 s with 50, 250, and 450 agents.

Figure 7 reports the response time, measured by one of the agents when: (i) all agents periodically make a request with a 10 s period, and (2) 50, 250, and 450 agents are present and concurrently accessing the cloud server. The *x*-axis represents the time since the beginning of the test, measured in seconds; the *y*-axis represents the time required to receive a reply from the server after calling the corresponding service. Since the whole test in Figure 7 lasts 300 s, each agent can make 30 requests during the selected time frame. To keep the total number of requests constant in all tests, the duration of the test is set to 30, 3, and 0.3 s, respectively, when the request period (i.e., the time between two subsequent requests of the same agent) is set to 1, 0.1, and 0.01 s.

The three curves correspond to the number of agents concurrently accessing the server: 50 agents (yellow), 250 (orange), 450 (blue).

At first glance, it is observable that the three curves, except for the amplitude, are similar. Situations when the response time is lower alternate with situations in which it is much higher. As expected, this becomes more evident when the number of agents increases. The curve corresponding to 50 agents is flatter than the curves corresponding to 250 and 450 (notice that the same peak is reached in the orange and the blue curve, due to unlucky circumstances in which many agents called a service simultaneously). All response times are below 1 s, which looks acceptable compared with the 10 s period according to which agents access the cloud (i.e., if 450 drones are transmitting their position every 10 s, the latter can be received with a 1-second delay, at worst). Specifically, the yellow curve is in the range of 0.15–0.23 s, with a peak of about 0.3 s. The orange curve is in the range of 0.15–0.95 s. Finally, the blue curve is in the range of 0.18–0.95 s.

**FIGURE 8:**
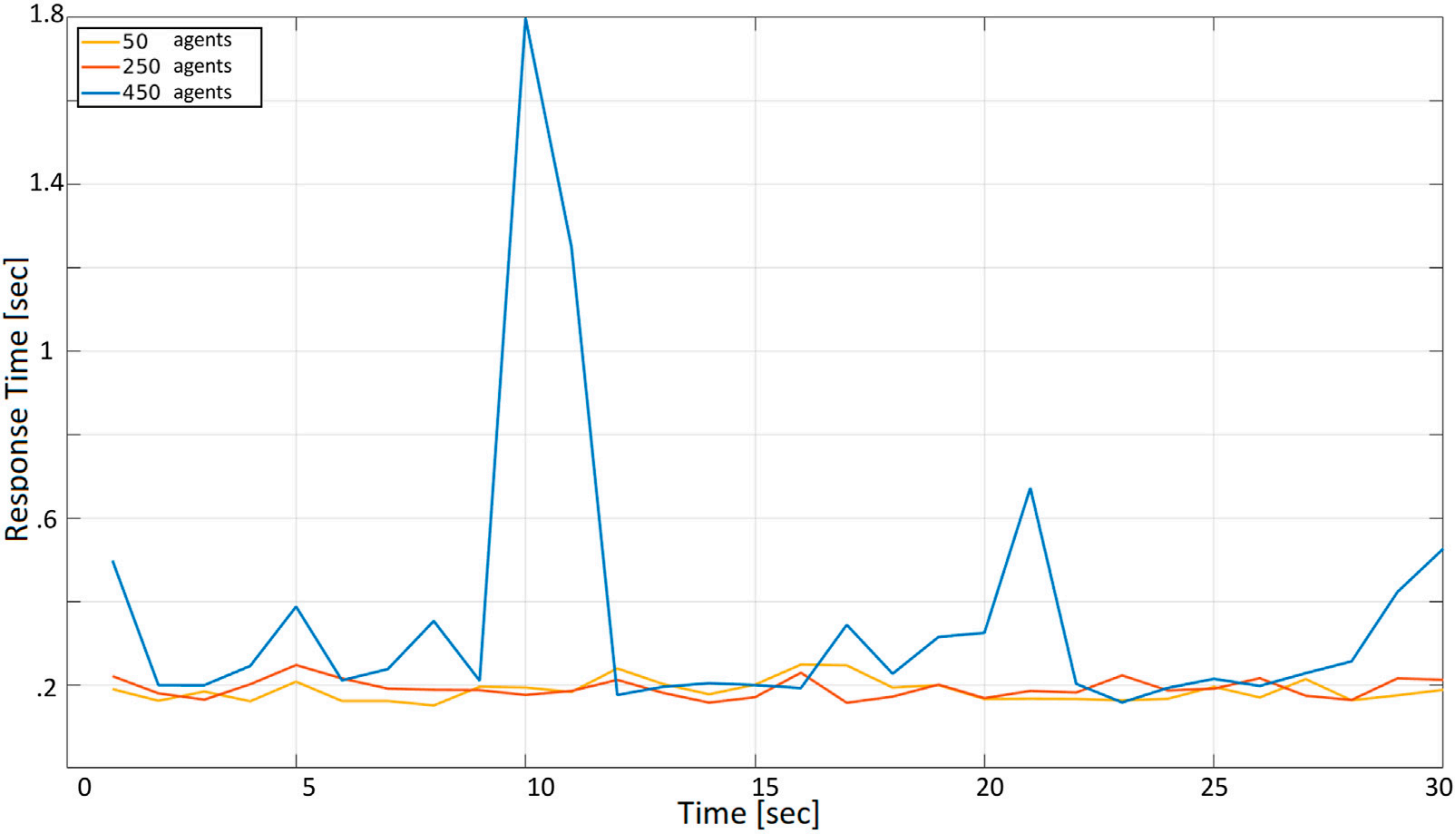
Cloud response time with a request period *r* = 1 s with 50, 250, and 450 agents.

In Figure 8 the request period is decreased to *r* = 1 s, and the difference between the yellow, orange, and blue curves is more evident. The yellow and orange curves exhibit an almost constant trend in the range between 0.18 and 0.28 s (i.e., the unlucky situation that led the orange curve to a 0.95 response time is not visible in this case). Instead, the blue curve is more affected by the decreased request period, with a maximum peak that reaches 1.8 s (i.e., if 450 drones are transmitting their position every second, this may be updated with a 1.8-second delay in the worst case).

**FIGURE 9:**
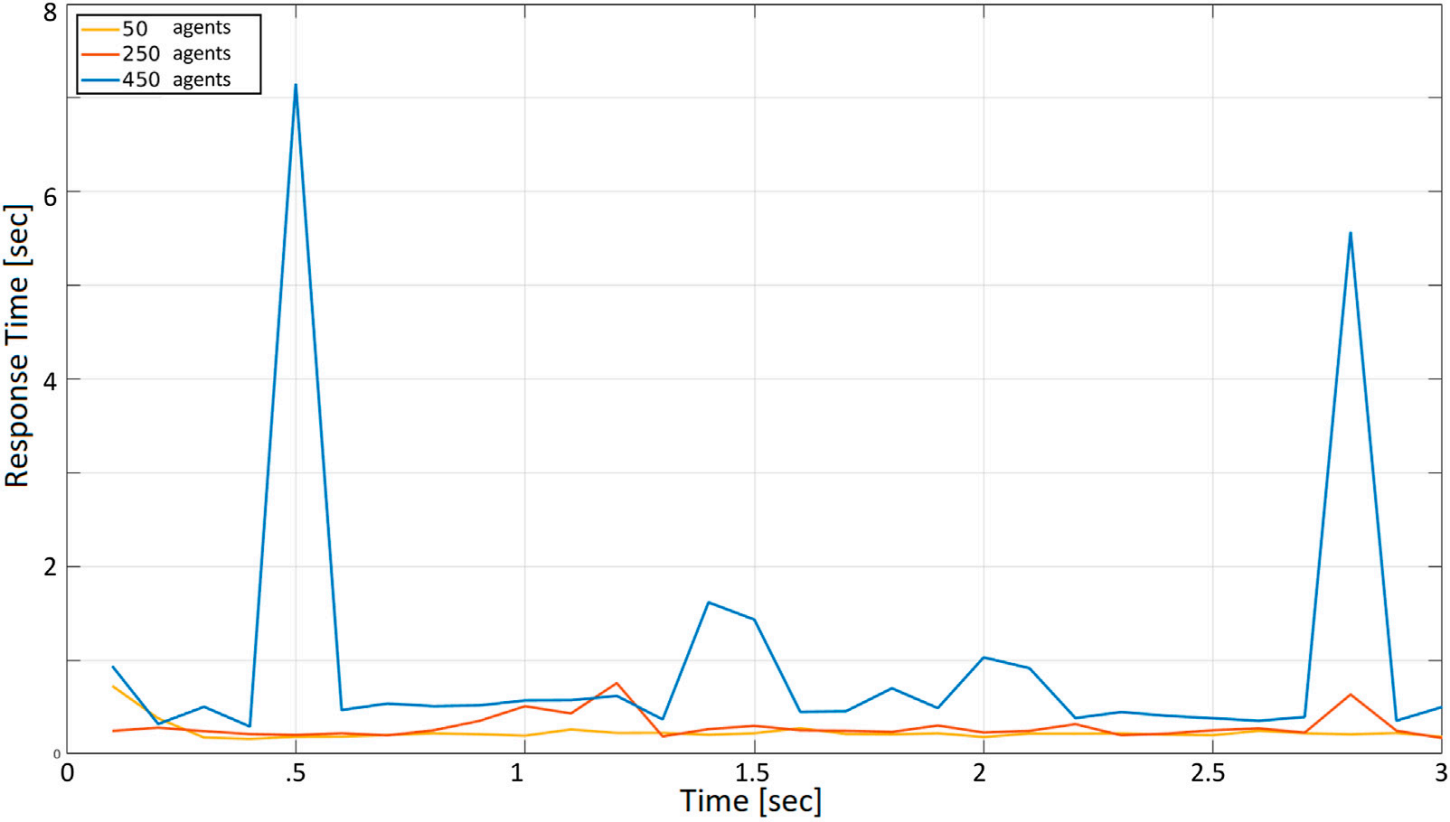
Cloud response time with a request period *r* = 0.1 s with 50, 250, and 450 agents.

A similar situation occurs when the period is *r* = 0.1 s, Figure 9, but this time the delays are much more consistent. The highest peaks of the blue curves are between 5.5 and 7 s, which are not compatible with a 0.1 request period. The orange curve is always above 0.2 s and reaches peaks above 0.7 s; similarly, the yellow curve response time is always above 0.2 s. These values are hardly compatible with the selected 0.1 request periods.

Table 1 contains the average values of the system response time, with additional values for the 150 and 350 agents cases.

**TABLE 1:** Average response times with different request periods and number of agents concurrently accessing the cloud.

		Number of agents (drones/residents)				
		**50**	**150**	**250**	**350**	**450**
Request period [sec]	**10**	0.1859	0.1915	0.1913	0.2148	0.3607
	**1**	0.1894	0.1946	0.2565	0.348	0.3939
	**0.1**	0.2329	0.2371	0.2893	0.6288	0.9744
	**0.01**	0.2271	0.3283	0.5454	0.9632	1.4379

“Request period” is the interval between two subsequent requests of the same agent; “Number of agents” are the number of drones or residents that periodically access the cloud during the test.

### 6.3 Path Computation

In the third phase, we measure the time required by the algorithm for path computation. Two different configurations are considered:

• same number of SCSs and targets;
• different number of SCSs and targets, but with a constant number of nodes in the graph.

The first test is performed by using the same number of SCSs and targets. Remember that we call “nodes” the set of all SCSs and targets: then, in the following, we measure the time the algorithm takes to solve a specific problem with an increasing number of nodes, equally split among SCSs and targets to be inspected. Please notice that we use the free academic version of the Gurobi software to carry out these tests (Python version): this version allows researchers to formulate problems with a maximum of 28 nodes for each optimization problem.

Table 2 summarizes the results with an increasing number of nodes: we start with four nodes and increase them after each test by adding two SCSs and two targets, up to the maximum number of nodes allowed by Gurobi. Then, for any given number of nodes, the algorithm is executed 10 times with different node coordinates, randomly computed: the average time required by the algorithm to find a solution and its standard deviation are computed over the ten runs. Finally, the procedure is repeated three times by imposing different inspection times when a drone is visiting a target: 60, 180, and 300 s.

**TABLE 2:** First test sumarized results.

		Insp. time = 60 s		Insp. time = 180 s		Insp. time = 300 s	
		Mean	Std.	Mean	Std.	Mean	Std.
N. of vertices	**4**	0.006	0.0038	0.0038	0.0013	0.0043	0.0016
	**8**	0.0311	0.0186	0.0317	0.0204	0.0244	0.012
	**12**	0.2611	0.1871	0.2723	0.1014	0.254	0.1502
	**16**	1.2698	2.5359	1.0459	0.8175	0.8675	0.5531
	**20**	5.3742	5.6554	5.811	6.7905	4.8461	4.0175
	**24**	39.6203	49.8196	32.2628	44.8377	48.058	53.0147
	**28**	67.0975	44.301	119.3972	95.8946	140.1556	119.2363

“N. of vertices” is the total number of targets and Social Charging Stations in the test; “Insp. Time” is the inspection time that drones spend on each target.

**FIGURE 10:**
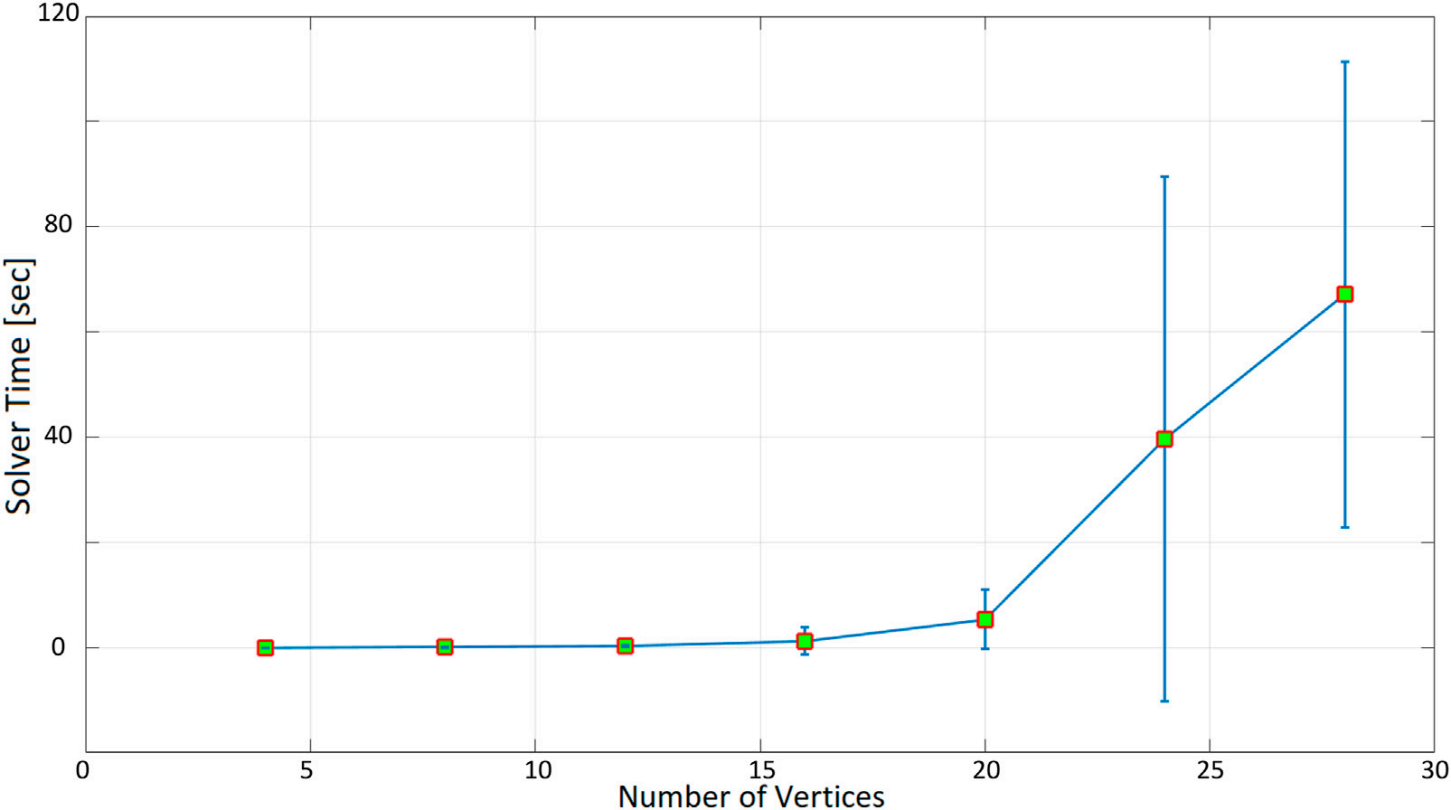
Optimization time (average and standard deviation) versus number of vertices, with the number of targets equal to the number of social charging stations, inspection time spent in targets equal to 60 s.

**FIGURE 11:**
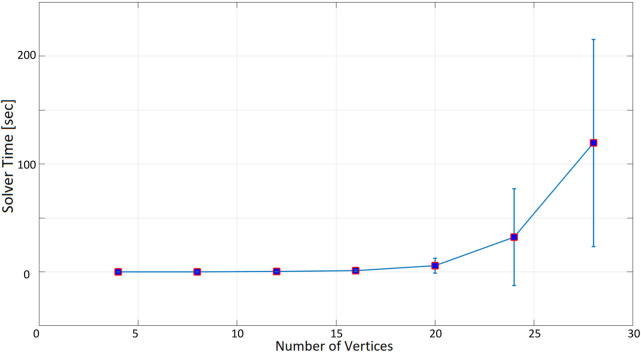
Optimization time (average and standard deviation) versus number of vertices, with the number of targets equal to the number of social charging stations, inspection time spent in targets equal to 180 s.

**FIGURE 12:**
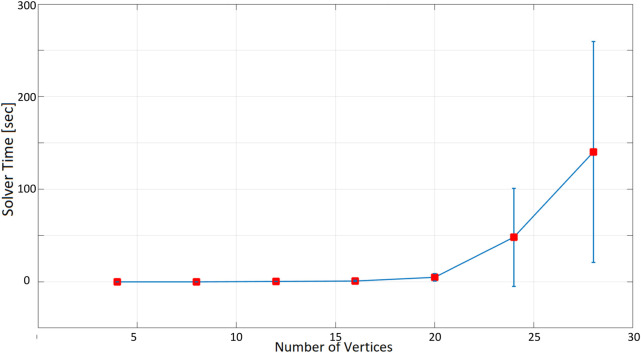
Optimization time (average and standard deviation) versus number of vertices, with the number of targets equal to the number of social charging stations, inspection time spent in targets equal to 300 s.

As expected, as the number of nodes increases, the time taken to find a solution increases exponentially. When the inspection time is 60 s (Figure 10), the computation time is in the order of tenths of a second up to 12 nodes. With 16 nodes, the optimization time increases to more than 1 s, and it is about 5 s for 20 nodes. The optimization time increases even more significantly when 24 and 28 nodes are considered, with an average of 40 and 67 s. In the 180-seconds and 300-seconds tests (Figures 11, 12), the situation is very similar to the 60-seconds test up to 20 nodes: a difference of a few seconds is detected when considering 24 nodes. However, the difference becomes dramatic when considering 28 nodes: an inspection time of 180 and 300 s per target yields an optimization time of, respectively, 119 and 140 s. As expected, also the standard deviation of the computation time increases as the number of nodes increases.

**FIGURE 13:**
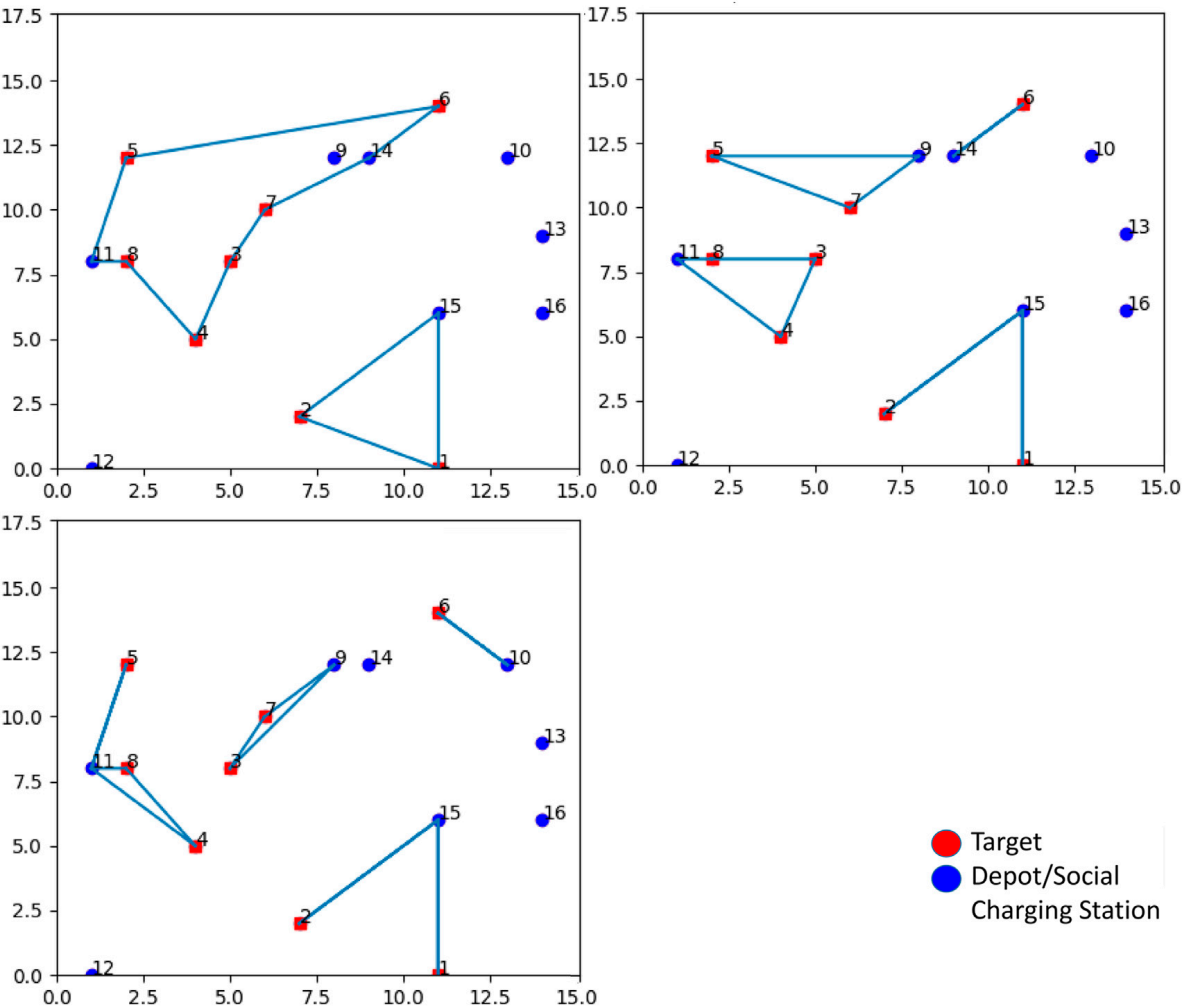
Top left: inspection time spent in targets equal to 60 s (Path 1: 11, 8, 4, 3, 7, 14, 6, 5, 11; Path 2: 15, 2, 1, 15). Top right: inspection time spent in targets equal to 180 s (Path 1: 14, 6, 14; Path 2: 9, 7, 5, nine; Path 3: 11, 8, 3, 4, 11; Path 4: 15, 2, 15, 1, 15). Bottom left: inspection time spent in targets equal to 300 s (Path 1: 10, 6, 10; Path 2: 9, 7, 3, 9; Path 3: 11, 5, 11, 8, 4, 11; Path 4: 15, 2, 15, 1, 15).

To summarize, setting a higher inspection time for each visited target requires a larger optimization time to find a solution. This happens because it is more difficult for the solver to find feasible solutions, as drones will be required to come back to an SCS for recharging more often. The resulting paths also confirm this evidence. Figure 13 show that, by increasing the inspection time, more SCSs need to be visited, and a higher number of closed paths are generated, up to the extent that, for some configurations, a solution cannot be found at all.

**FIGURE 14:**
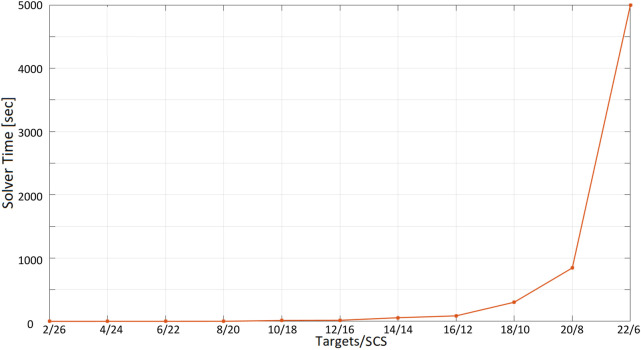
Optimization time (average and standard deviation) with 28 vertices as the ratio number of targets/number of Social Charging Stations varies, inspection time spent in targets = 60 s.

The second test performed involves a different number of SCSs and targets (with a constant number of nodes): we report the optimization time versus the ratio “number of target”/“number of SCSs.” The test is conducted with a 60-second inspection time when visiting targets, starting from the ratio 2/26 (2 targets and 26 SCSs) to 22/6 (22 targets and 6 SCSs), increasing the number of targets while decreasing the number of SCSs by two units at each iteration. We require the total number of nodes to be 22 + 6 = 28 because of the limitations of Gurobi free version (and because the optimization time with this last configuration requires more than an hour and a half to find a solution: a more powerful computer providing higher performances might be used). For each ratio “number of target”/“number of SCSs,” 10 randomly computed configurations of nodes are considered by computing averages and standard deviations of the optimization time. Results are shown in Table 3. From Figure 14, it can be observed that a solution is found in less than 2 s up to the ratio 8/20. After this point, the optimization time starts to increase considerably up to the value of 5000 s for the ratio 22/6.

**TABLE 3:** Second test summarized results.

		Inspection time = 60 s	
		Mean	Std.
Ratio =Target/SCS	***r* = 2/26**	0.0214	0.0027
	***r* = 4/24**	0.1494	0.0651
	***r* = 6/22**	0.5036	0.2759
	***r* = 8/20**	1.0784	0.2501
	***r* = 10/18**	14.1215	26.7053
	***r* = 12/16**	17.1159	14.3806
	***r* = 14/14**	57.2587	37.0916
	***r* = 16/12**	87.1039	97.2958
	***r* = 18/10**	302.2895	358.7991
	***r* = 20/8**	844.6466	1.02E + 03
	***r* = 22/6**	Over 5000	—

“Ratio = Target/SCS” is the ratio between the number of targets and Social Charging Stations in the test; “Insp. Time” is the inspection time that drones spend on each target.

**FIGURE 15:**
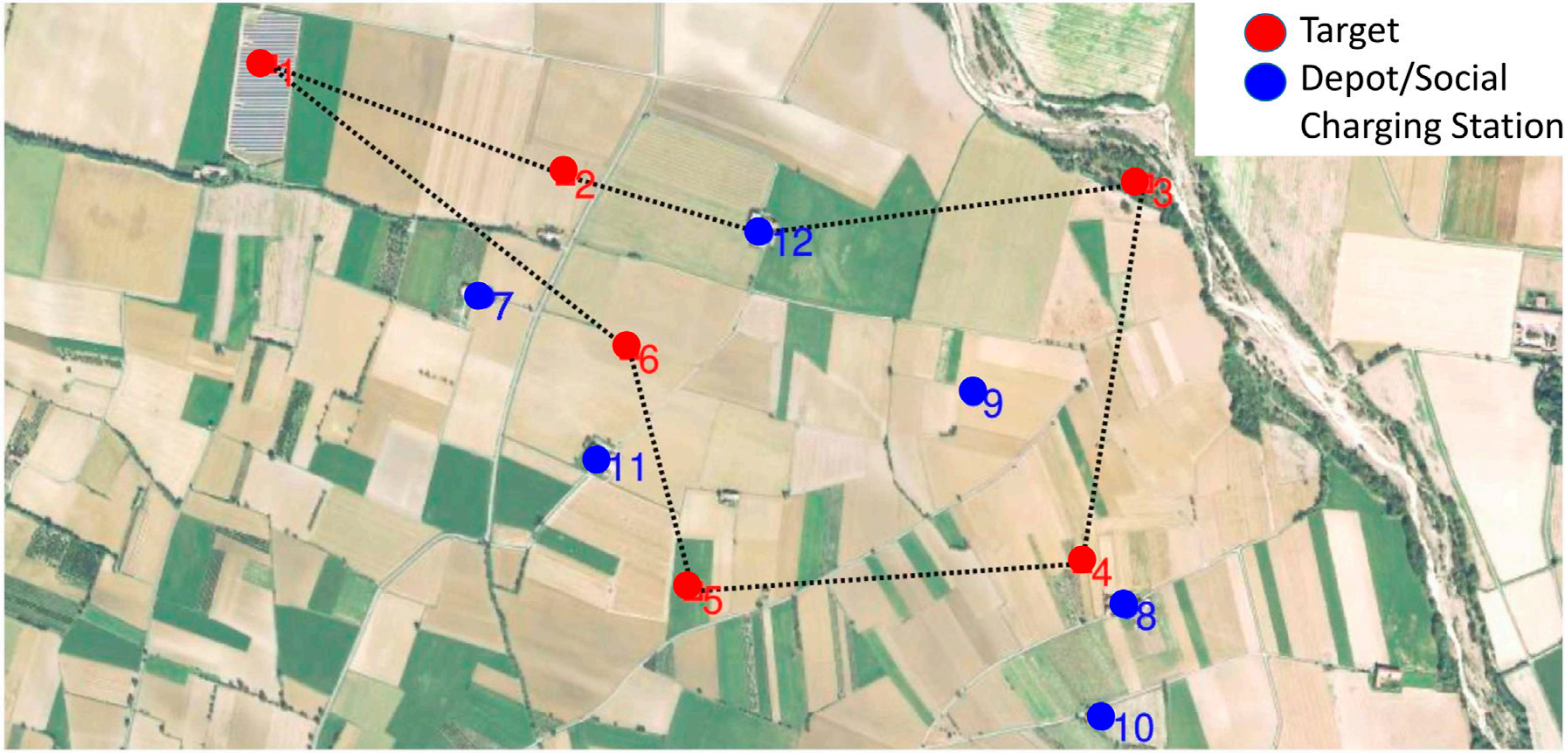
Geo-referenced path in a real world scenario superimposed to a Google Earth image.

Finally remind that, up to now, the nodes are randomly positioned. In the case that this algorithm had to be used in a real situation, the targets should be chosen according to inspection needs, and SCSs would be positioned at the residents volunteers’ houses coordinates. Figure 15 is an example of how paths could look like in a real-life situation. Targets are highlighted in red: in this case they correspond to photo-voltaic plants to be periodically monitored for maintenance (target 1 on the top left of the image), or fields where the dryness of the vegetation should be checked (other targets). SCSs are constrained to be close to houses.

## 7 Conclusion

In this article, we address the problem of pre- and post-event monitoring and assessment with UAVs in emergency scenarios. To this end, we propose the concept of Social Drone Sharing (SDS): citizens are involved in the monitoring process through Social Charging Stations (SCS) to extend the autonomy range of drones (Morando et al., 2020).

The following results have been achieved in the present work.

• A cloud-based software architecture has been developed that allows communication between the actors involved in the inspection mission: the Civil protection, the resident volunteers that make their properties available as SCSs, and the drones themselves. Each actor can perform certain actions to exchange information through the services provided by a cloud server and the related database. Tests show that, from a qualitative point of view, the control flow when accessing services is correct: no errors were reported during the execution of the tests. From a quantitative perspective, the choice to implement services on the cloud (Microsoft Azure) has been evaluated through systematic tests to measure the server’s response time with an increasing request rate and a number of agents.
• An optimization algorithm has been implemented inspired from (Sundar et al., 2016), which allows finding the optimal paths for multiple drones in the presence of multiple targets to inspect and SCS. Tests have been performed to evaluate the time required to find a solution with an increasing number of nodes, increased inspection time when visiting targets, and finally, a different ratio between the number of targets and the number of SCS available for recharging.

As a future work, we are going to consider the following lines of research.

• We will ensure that the system can handle various models of drones with different characteristics, e.g., speed and battery consumption. For laboratory experiments, the system can currently handle only one type of drone, the DJI Tello, which is a great solution for indoor experiments but is entirely useless in a realistic outdoor scenario.
• The optimization model returns a set of closed paths, as the drones have to depart and return to the same point. However, this is not necessarily true in our scenario, where a drone could be instructed to complete its path by landing at an SCS that is different from the starting one. The optimization problem is going to be modified and updated accordingly.
• In the current optimization model, every SCS is assumed to have a drone ready to take off: i.e., SCS are considered depots, not only recharging stations. These two concepts should be decoupled since SCS may exist that do not have any drone ready to take off when optimization starts. This problem has been temporarily solved by providing the Civil protection with the set of paths found and then asking them to assign drones to paths and select depots manually. This solution makes sense as a prototype to demonstrate the SCS principle. Still, it may not be appropriate in real cases. Once again, the optimization problem is going to be modified and updated accordingly.

Once these aspects will be addressed and the whole system refined, we aim to start a larger trial with the support of mountain communities in the North of Italy.

## Data Availability

The raw data supporting the conclusion of this article will be made available by the authors, without undue reservation.
